# Researches on the Neuralgiæ Treated by Quinine and Its Preparations

**Published:** 1846-08

**Authors:** 


*Researches on the Neuralgiæ treated by Quinine and its Preparations. By Dr. Hermel.*—The numerous cases observed by M. Hermel have led him to the following conclusions:

1. There are essential or idiopathic neuralgias which effect the intermittent or remittent type. These neuralgias are treated with success by quinine and its preparations.

2. There are neuralgias which appear with a febrile action, and either follow rigors or flushings of heat, and which are analogous to one of the stages of intermittent fever. These, like the preceding, are also cured by the employment of the antiperiodic specific.

3. There are diseases which present a greater or less number of different affections during their course. Such are gout and scrofula. When one of these sympathetic affections has just disappeared, such for example, as hemorrhoids, and neuralgia appears, the best, and indeed the only method of curing it, even if it have the intermittent type, is to re-establish the affection by which it was preceded. These neuralgias do not require quinine.

4. In neuralgias with an intermittent type, which are symptomatic of a disease or an affection which does not present this type, quinine and its preparations may be employed, as an accessory, to combat the intermittent nervous pain if it persists.

5. We ought not to count on the modification of the first attacks after the first doses of the medicine, especially in the essential intermittent neuralgias.

6. Intermittent neuralgias, like fevers of a similar type, are liable to return. It is necessary, therefore, to continue the administration of quinine and its preparations, not only after the disappearance of all the symptoms, but during the period of about seven years (*septénaire.*)—*Ibid, from Gazette Médicale.*

